# Objective quantification of homophily in children with and without disabilities in naturalistic contexts

**DOI:** 10.1038/s41598-023-27819-6

**Published:** 2023-01-17

**Authors:** Chitra Banarjee, Yudong Tao, Regina M. Fasano, Chaoming Song, Laura Vitale, Jue Wang, Mei-Ling Shyu, Lynn K. Perry, Daniel S. Messinger

**Affiliations:** 1 Department of Psychology, University of Miami, Coral Gables, FL USA; 2 Department of Electrical & Computer Engineering, University of Miami, Coral Gables, FL USA; 3 Department of Physics, University of Miami, Coral Gables, FL USA; 4 Department of Psychology, University of Science and Technology of China, Hefei, China

**Keywords:** Human behaviour, Social behaviour

## Abstract

Homophily, the tendency for individuals to preferentially interact with others similar to themselves is typically documented via self-report and, for children, adult report. Few studies have investigated homophily directly using objective measures of social movement. We quantified homophily in children with developmental disabilities (DD) and typical development (TD) using objective measures of position/orientation in preschool inclusion classrooms, designed to promote interaction between these groups of children. Objective measurements were collected using ultra-wideband radio-frequency tracking to determine social approach and social contact, measures of social movement and interaction. Observations of 77 preschoolers (47 with DD, and 30 TD) were conducted in eight inclusion classrooms on a total of 26 days. We compared DD and TD groups with respect to how children approached and shared time in social contact with peers using mixed-effects models. Children in concordant dyads (DD-DD and TD-TD) both moved toward each other at higher velocities and spent greater time in social contact than discordant dyads (DD-TD), evidencing homophily. DD-DD dyads spent less time in social contact than TD-TD dyads but were comparable to TD-TD dyads in their social approach velocities. Children’s preference for similar peers appears to be a pervasive feature of their naturalistic interactions.

## Introduction

Interactions with peers in early school settings are essential for social skill development including reciprocity, self-regulation, and language mediated interaction^1–3^. Children with developmental disabilities—spanning adaptive, cognitive, socio-communicative, emotional, and/or motor development delays of mixed etiology^4^—exhibit a range of difficulties with peer interaction^5^. After 3 years of age, inclusion classrooms, in which children with developmental disabilities (DD) are educated alongside typically developing (TD) children, are the only government mandated form of intervention for children with disabilities^6^, making these classrooms a critical developmental context for children with DD. It is, however, naïve to think that children’s mere presence together in a classroom indicates that they interact. There is evidence that children preferentially associate with similar children, a phenomenon known as homophily^7–9^. However, this evidence primarily comes from subjective ratings from teachers. There is little research exploring objectively measured patterns of social interaction among children with DD and TD peers in naturalistic settings (but see^10,11^). We reasoned that the velocity with which children approached one another was an objective index of their attraction to specific peers while their time in social contact with other children peers was an objective index of their preference for those peers. Thus, the movement metrics of social approach, how children move toward one another, and social contact, how children remain proximal to one another, afford an objective view of their social interaction^12^.

Teacher report in inclusive settings indicates that children with disabilities are positively impacted by being educated alongside TD peers^13^. However, these benefits may be limited by the tendency of children to preferentially interact with more similar peers. Homophily is the tendency of individuals to interact preferentially with peers who are similar to themselves. Using teacher ratings of children’s play interaction frequency, Chen et al. added to growing evidence of homophily effects in the preschool classroom, finding that children in concordant dyads (DD-DD, TD-TD) were more likely to engage in play interactions than children in discordant dyads (DD-TD)^14^. More generally, findings of homophily with respect to language ability, gender, and academic performance indicate that children are more likely to interact with others who share qualities most similar to themselves^7–9^.

Previous studies of homophily in inclusion classrooms are primarily based on teacher-reports (and self-reports) of interaction frequency, neither of which objectively capture ongoing dyadic movements between individuals. Thus, the tendency of children to interact with children similar to themselves has been evidenced via, predominantly, teacher reports of children’s interaction patterns. However, teacher report may be biased by individual characteristics of the reporting teacher, the classroom context, as well as by child characteristics^15,16^. Quantifying social movement through social approach and social contact provides the opportunity to objectively measure interaction in naturalistic, inclusive settings aimed at promoting social development for both children with and without disabilities.

Objective location tracking in inclusion classrooms is an emerging method for investigating the degree of social interaction between DD and TD children. Fasano et al., for example, used automated analyses of audio recordings in a subset and children examined here. Children tended to speak to other children in the same disability category (TD children and children with ASD and DD)^17^. But Fasano et al. did not examine the behavioral primitives of social approach and social contact that are the bases of the indices of homophily examined in the current larger sample. Here, we examine children’s tendency to move toward and to be with other children. We operationalize homophily as higher approach velocities and a greater proportion of time in social contact in concordant dyads than in discordant dyads.

A specific category of developmental disability, autism spectrum disorder (ASD), is defined in part by deficits in social communication that extend to atypical social approach. Previous investigations have examined social approach in children with ASD using manually coded movement toward or away from peers^18^. The frequency of manually coded interactions in a preschool inclusion classroom did not indicate differences in latency to engage in interaction between children with ASD and their TD peers^19^. However, human observers cannot simultaneously measure the social engagement, movement, and orientation of every child in a classroom. We measured social approach and social contact objectively to investigate differences and similarities among children with ASD, children with DD, and TD children with neither disability.

Human measurement limits the possibility of collecting large-scale datasets that characterize children with ASD, DD, and TD in naturalistic settings^20^. Compared to human observers, objective measurement affords increases in measurement efficiency and precision that allow for analyses of big behavioral data^14,21,22^. Here, off-the-shelf sensors were used to record children’s location and orientation during naturally occurring classroom interactions yielding an average of over eight and half hours of observation per child.

Ultra-wideband (UWB) positioning has been used to characterize human velocity in a variety of indoor contexts, using time of arrival (ToA) algorithms to track location^23^. UWB radio frequency identification (RFID) tracks the location of active tags worn by multiple participants in complex environments^24–26^. UWB RFID has been validated for tracking children’s movement in the preschool classroom^24^, and used to conduct location-tracking in a general education kindergarten classroom^8^. In the current study, children wore two tags, which provided information on orientation and location, to compare the movement of children with DD, their TD peers in a naturalistic context, the preschool classroom. Based on previous findings^8,13,14^, we predict homophily will be evident in patterns of social approach and social contact in inclusive settings, in that dyads of children similar to themselves (ASD-ASD, DD-DD, TD-TD) will approach at higher velocities and spend more time in social contact.

## Results

### Social approach

**Figure 1:**
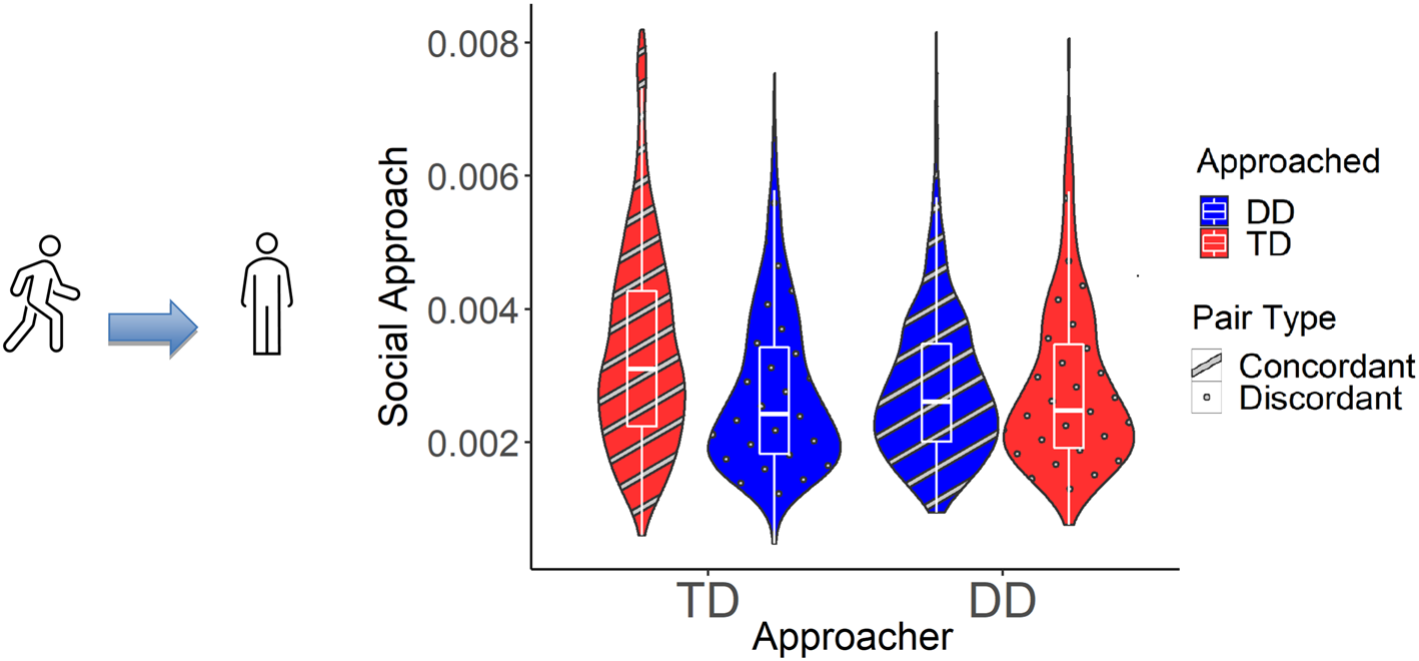
Social approach. Social approach is the mean proportion of the distance between a pair of children traversed by the Approacher per tenth of a second. Approacher is the child who is moving and Approached is the child who is being approached. When Approacher and Approached are both DD or both TD, the pair type is concordant. DD includes both ASD and O/DD. Children in concordant dyads (DD-DD and TD-TD) approached one another at higher velocities than discordant dyads (TD-DD) (*p*s < 0.001). Children with DD were approached by other children at lower velocities than TD children (*p* = 0.038).

Social approach is operationalized as the velocity with which one child directly approached another expressed as a proportion of their initial distance. Children in concordant dyads (DD-DD and TD-TD) approached one another at higher velocities than discordant dyads (TD-DD) (*p*s < 0.001, see Table 1 and Fig. 1). Analyses did not indicate overall differences in the velocity with which DD and TD children approached others in the classroom (see Supplementary Table 2). However, DD children were approached by other children at lower velocities than TD children (*p* = 0.038). Control analyses indicated that children with DD did not differ from TD children in a non-social (individual) measure of movement (*p* = 0.754), linear velocity, the overall speed at which children move in the classroom (Supplementary Table 5).

**Table 1:** Social approach velocities.

Predictors	Social approach					
	*B*	SE	CI	*t*	*p*	*d*
(Intercept)	0.00314	0.00023	0.00269–0.00359	13.66	< 0.001	
Approached [DD]	− 0.00009	0.00004	− 0.00017 to − 0.00000	− 2.07	**0.038**	− 0.08
Homophily [concordant]	0.00020	0.00004	0.00012–0.00029	4.87	** < 0.001**	0.18
Random effects						
σ^2^	0.00000					
Child	0.00000					
Classroom	0.00000					
ICC	0.30082					
Observations	3108					

σ^2^—residual variance at level 1 (observation). ICC—intraclass correlation. Social approach is the mean proportion of the distance between a pair of children traversed by the Approacher per tenth of a second. Approached [DD] compares velocities at which children with DD were approached to velocities at which children with TD were approached. Negative coefficients indicate that children with DD were approached at lower velocities than TD children.

Significant values are in bold.

**Figure 2:**
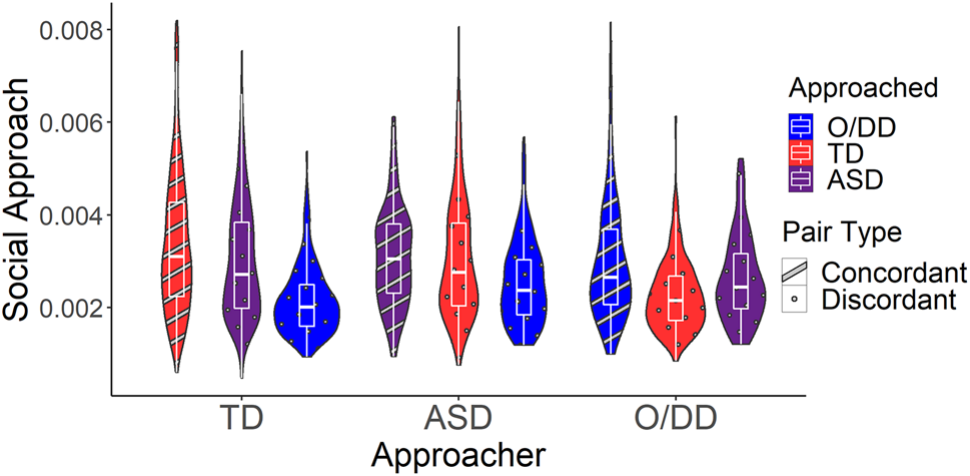
Social approach (disaggregated). This figure disaggregates the DD group into O/DD and ASD. Social approach is the mean proportion of the distance between a pair of children traversed by the Approacher per tenth of a second. Approacher is the child who is moving, and Approached is the child who is being approached. When Approacher and Approached are both DD, both TD, or both ASD, the pair type is concordant. Concordant dyads (ASD-ASD, O/DD-O/DD, TD-TD) approached one another more quickly than discordant dyads (*p* < 0.001). Children with ASD and O/DD did not differ in how they were approached by other children.

A subset of children with DD were categorized as having ASD. That is, the full DD group (*n* = 47) could be disaggregated into an ASD group (*n* = 24) and another DD (O/DD) group (*n* = 23). ASD is a type of DD characterized by pervasive social interaction difficulties and repetitive behaviors. Parallel analyses disaggregating DD into ASD and O/DD also yielded strong homophily effects (*p*s < 0.001). Concordant dyads (ASD-ASD, O/DD-O/DD, TD-TD) approached one another more quickly than discordant dyads (see Table 2 and Fig. 2).

**Table 2:** Social approach disaggregated (TD, ASD, O/DD).

Predictors	Social approach					
	*B*	SE	CI	*t*	*p*	*d*
(Intercept)	0.00314	0.00023	0.00269–0.00358	13.78	< 0.001	
Approached [ASD]	− 0.00007	0.00005	− 0.00017 to 0.00003	− 1.36	0.175	− 0.05
Approached [O/DD]	− 0.00002	0.00006	− 0.00014 to 0.00010	− 0.33	0.742	− 0.01
Homophily [concordant]	0.00022	0.00004	0.00013–0.00031	4.99	** < 0.001**	0.19
Random effects						
σ^2^	0.00000					
Child	0.00000					
Classroom	0.00000					
ICC	0.29761					
Observations	3108					

σ^2^—residual variance at level 1 (observation). ICC—intraclass correlation. Social approach is the mean proportion of the distance between a pair of children traversed by the Approacher per tenth of a second. Approached [ASD], and Approached [O/DD] compares velocities at which children with ASD and children with other (unspecified) DD, respectively, were approached to velocities at which children with TD were approached. Negative coefficients indicate that children with ASD and O/DD were approached at lower velocities than TD children.

Significant values are in bold.

### Social contact

**Figure 3:**
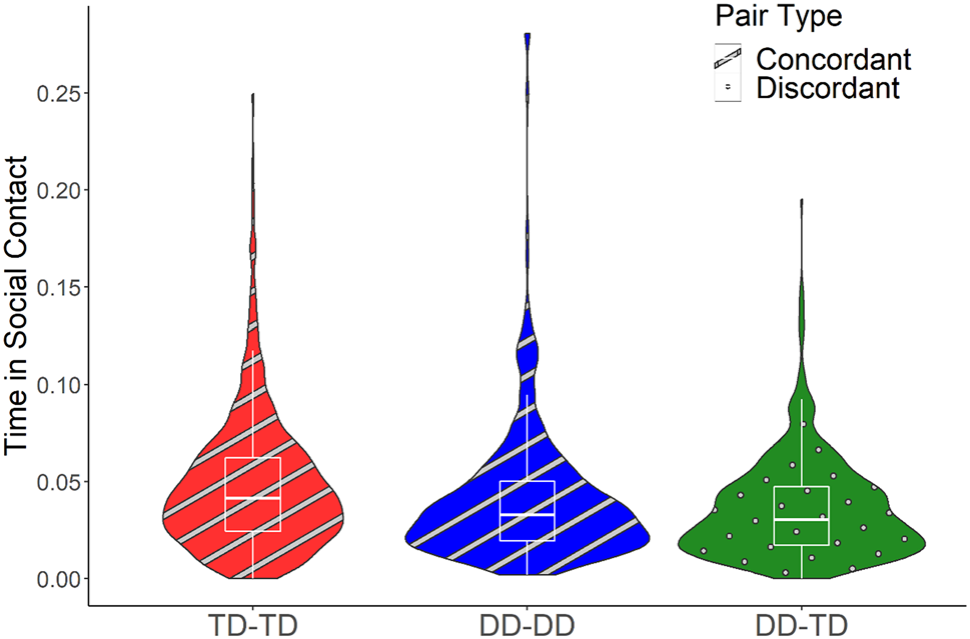
Social contact. Time in social contact is the proportion of time a pair of children were in social contact divided by the time both children were present in the classroom. Social contact is between 0.2 and 2 m and mutual relative orientation < 45°. DD includes both ASD and O/DD. Children in concordant dyads (DD-DD/TD-TD) spent a greater proportion of time in social contact than discordant dyads (DD-TD, *p* < 0.001), and DD-DD dyads were in social contact less than TD-TD dyads (*p* < 0.001).

Social contact occurred when two children fulfilled both a distance criterion (children were within 0.2 and 2 m as established by the radial distribution function), and an interpersonal orientation criterion (each was within 45° of face-to-face interaction). Social contact is expressed as a proportion of the time in which two children were both present in the classroom. Children in concordant dyads (DD-DD/TD-TD) spent a greater proportion of time in social contact than discordant dyads (DD-TD, *p* < 0.001). In addition, DD-DD were in social contact less than TD-TD dyads, as revealed by a significant interaction term (*p* < 0.001). Children with DD did not differ from TD children in the overall time in social contact with other children (*p* = 0.987, see Table 3 and Fig. 3).

**Table 3:** Social contact.

Predictors	Time in social contact					
	*B*	SE	CI	*t*	*p*	*d*
(Intercept)	0.04	0.00	0.04–0.05	13.22	< 0.001	
[DD]	0.00	0.00	− 0.00 to 0.00	0.02	0.987	0.00
Homophily [concordant]	0.01	0.00	0.01–0.01	6.63	** < 0.001**	0.24
[DD] x Homophily [concordant]	− 0.01	0.00	− 0.02 to − 0.01	− 4.29	** < 0.001**	− 0.16
Random effects						
σ^2^	0.00					
Child	0.00					
Classroom	0.00					
ICC	0.08					
Observations	3108					

σ^2^—residual variance at level 1 (observation). ICC—intraclass correlation. Time in social contact is the time the pair were in social contact divided by the time both children were present in the classroom. The [DD] term compares the proportion of time in social contact for children with DD with time in social contact for children with TD. The Homophily term indicates that children spent greater proportion of time in social contact when interacting with a concordant partner, compared to a discordant partner. The interaction term, signified by x, indicates that children with DD spent less time in social contact with other DD children, compared to TD-TD dyads. DD includes both ASD and O/DD.

Significant values are in bold.

**Figure 4:**
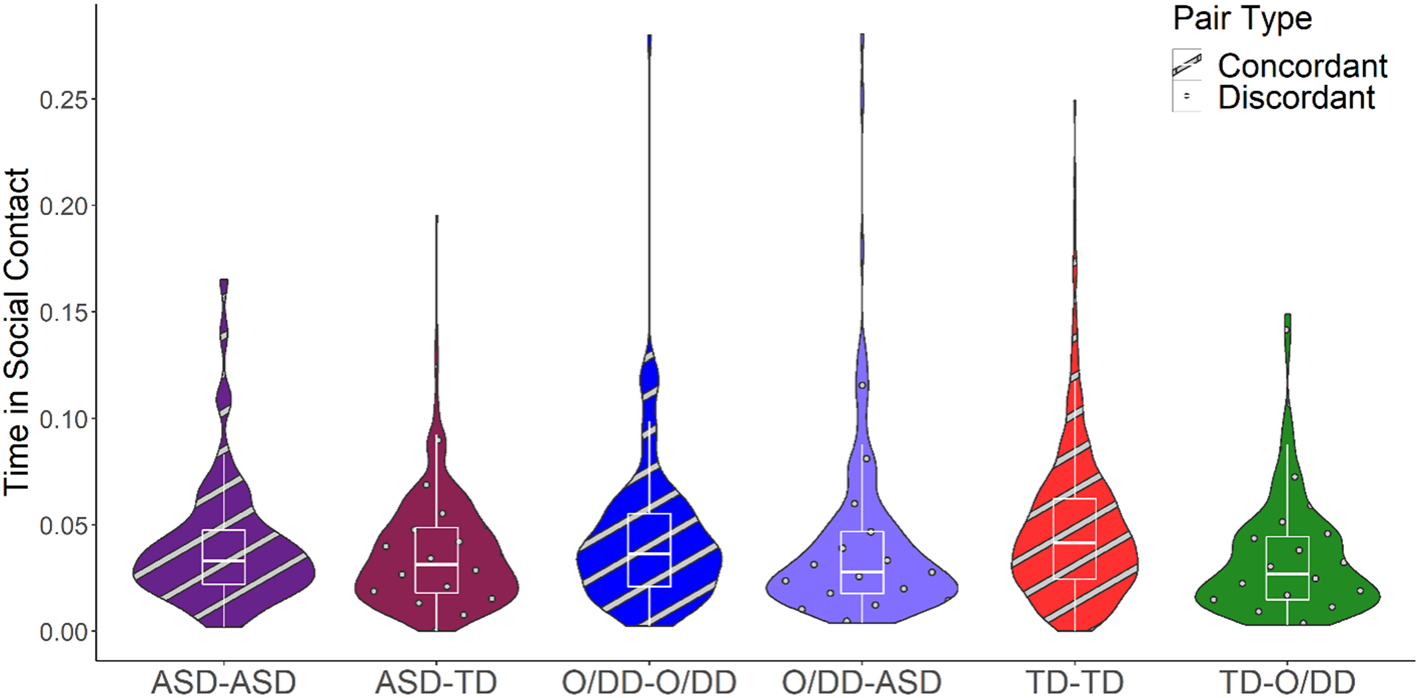
Social contact (disaggregated). Time in social contact is the proportion of time a pair of children were in social contact divided by the time both children were present in the classroom. This figure disaggregates the DD group into O/DD and ASD. Social contact is the proportion of time in social contact out of the total time shared with each individual peer. Social contact is between 0.2 and 2 m and mutual relative orientation < 45°. Children in concordant dyads (O/DD-O/DD/ASD-ASD/TD-TD) spent a greater proportion of time in social contact than discordant dyads (DD-TD, *p* < 0.001), and O/DD-O/DD (p < 0.001) and ASD-ASD (*p* = 0.014) dyads were in social contact less than TD-TD dyads.

Disaggregated parallel analyses separating the DD group into ASD and O/DD replicated this result, exhibiting homophily effects and interactions between homophily and children with ASD and O/DD (see Table 4 and Fig. 4). Children with ASD, O/DD, and TD children did not differ in time in social contact. However, concordant dyads with developmental disabilities (ASD-ASD and O/DD-O/DD) spent less time in social contact than TD-TD dyads.

**Table 4:** Social contact disaggregated (TD, ASD, O/DD).

Predictors	Time in social contact					
	*B*	SE	CI	*t*	*p*	*d*
(Intercept)	0.04	0.00	0.04–0.05	13.21	** < 0.001**	
[O/DD]	0.00	0.00	− 0.00 to 0.01	1.23	0.218	0.10
[ASD]	− 0.00	0.00	− 0.01 to 0.00	− 0.76	0.444	− 0.09
Homophily [concordant]	0.01	0.00	0.01–0.01	5.83	** < 0.001**	0.96
[O/DD] x Homophily [concordant]	− 0.01	0.00	− 0.02 to − 0.01	− 4.00	** < 0.001**	− 0.65
[ASD] x Homophily [concordant]	− 0.01	0.00	− 0.01 to − 0.00	− 2.47	**0.014**	− 0.44
Random effects						
σ^2^	0.00					
Child	0.00					
Classroom	0.00					
ICC	0.08					
Observations	3108					

σ^2^—residual variance at level 1 (observation). ICC—intraclass correlation. Time in social contact is the time the pair were in social contact divided by the time both children were present in the classroom. The [ASD] and [O/DD] terms compare the proportion of time in social contact for children with ASD and other DD, respectively, with time in social contact for children with TD. The Homophily term indicates that children spent greater proportion of time in social contact when interacting with a concordant partner, compared to a discordant partner. The interaction term, signified by x, indicates that both children with ASD and O/DD spent less time in social contact with other ASD and O/DD children, compared to TD-TD dyads.

Significant values are in bold.

### The association of social approach and social contact

**Figure 5:**
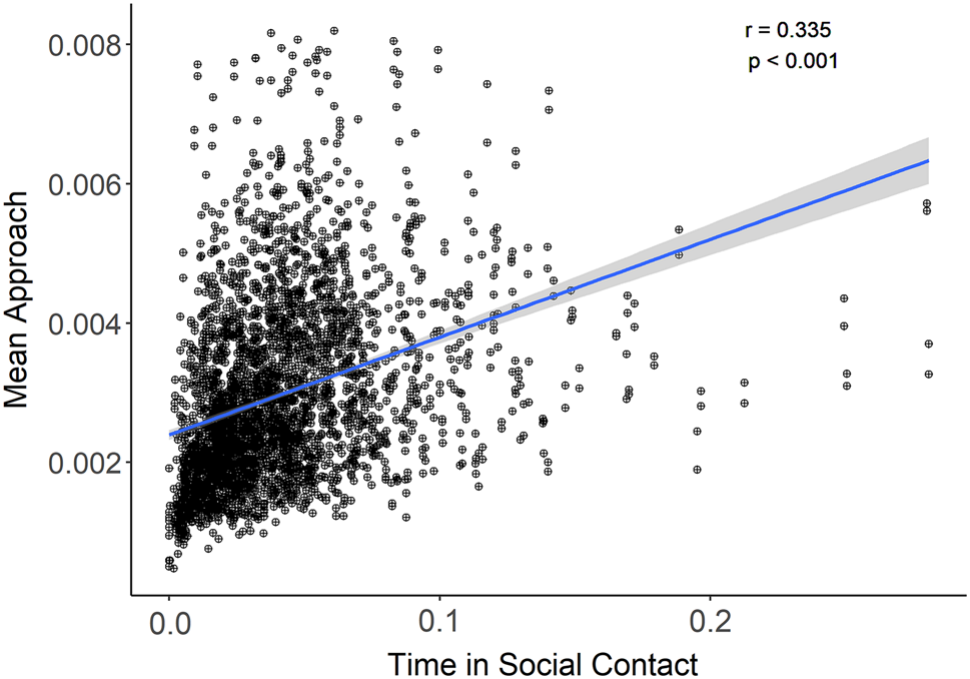
The association between social approach and social contact. Mean social approach values on the y-axis by time in social contact on the x-axis for all pairs of children for all observations. Pairs who approached each other at higher velocities spent more time in social contact over the course of the classroom day. The correlation was tested using Pearson’s correlation coefficient. Social approach is the mean proportion of the distance between a pair of children traversed by the Approacher. Time in social contact is the time the pair were in social contact divided by the time both children were present in the classroom.

Social approach velocities and social contact were associated (Pearson’s correlation coefficient r = 0.335, *p* < 0.001, see Fig. 5).

## Discussion

Homophily effects, the tendency to interact preferentially with those similar to oneself, were evident in both social approach and social contact. Children with (*n* = 47) and without (*n* = 30) disabilities approached children similar to themselves (i.e., DD-DD and TD-TD) at higher velocities than dyads in which one child had a disability and the other did not (TD-DD). Similarly, DD-DD and TD-TD dyads spent a greater proportion of time in social contact than TD-DD dyads.

We surmise that greater velocity of movement toward peers characterizes preference for those peers. Children’s velocity of approach indexes their motivation to be close to or avoid specific other children. Social contact is a transparent measure of sociality. The current measure of approach is entirely data-driven in that it uses no a priori criteria for what does and does not qualify as approach. Rather approach is a continuous measure characterized as *all* movement that brings child A toward child B. By contrast, our measure of social contact is a categorical measure that uses an alternative method of strict data-driven criteria to label social (vs non-social) interaction: social contact occurs when the radial distribution function exceeds unity and the partners are within 45° of face-to-face orientation. Although social approach and social contact are independent constructs, they are nevertheless related. Pairs of children who approached each other at higher velocities spent more time in social contact (see Fig. 5). The moderate association between social approach and social contact suggests the utility of these measures for indexing children’s social preferences.

Investigating homophily in both social approach and social contact highlighted the role of the dyad. Social approach involves both a child who approaches and a child being approached. Children with DD did not differ from TD children in the velocities at which they approached other children. However, children with DD were approached at lower velocities than TD children. The results indicate that it is imperative to distinguish who is approaching whom when investigating agents who differ from one another such as children with and without developmental disabilities. Both children with DD and TD children approached similar partners (DD-DD, TD-TD) at higher velocities than dissimilar partners (TD-DD). Similarly, social contact is defined by a child and a partner. However, children with DD did not differ from TD children in their individual levels of social contact. Instead, pairs of children with DD were in social contact with each other less than pairs of TD-TD children. Patterns of homophilic social interaction in approach velocities and time in social contact indicate that children with TD and DD face barriers interacting with one another in inclusion classrooms.

Of note, there was no evidence that DD differences in social movement were attributable to DD differences in overall velocity. There were no differences in the linear velocity (overall speed) of children with DD and their TD peers. Thus, the overall pattern of results suggests that the observed social approach effects are not a characteristic of DD and TD children but rather of their dyadic interaction. Higher velocities of approach in concordant dyads may be interpreted as smoother and more coordinated patterns of movement.

The sample of preschoolers with DD included a subsample of children with ASD, a disorder characterized by difficulties in social communication and approach^27,28^. Analyses disaggregating DD into ASD and other developmental disabilities (O/DD), further specified the homophily effect. That is children with ASD, children with O/DD, and TD children moved toward children in the same group at higher velocities than they moved toward children in different groups (Table 2). This implies that individuals with ASD and other developmental disabilities distinguish one another in inclusion classrooms. Likewise, supplementary analyses of social contact further distinguished homophily effects in children with ASD and O/DD (Table 4).

Children with ASD, children with O/DD, and TD children all spent proportion more time in social contact with children in the same grouping than with children in other groupings. It was striking that children with ASD did not show lower levels of social contact with peers overall, given that this is a defining characteristic of the disorder. Instead, both ASD-ASD dyads and O/DD-O/DD dyads spent less time in social contact than TD-TD dads. These homophily results are reminiscent of reports indicating that adults with ASD report preferring to interact with other adults with ASD, while TD adults report preferring interactions with other TD individuals^29^.

There are several limitations to this study. First, no information was available on children’s activities outside of school. If children socialized outside of the classroom this may have influenced patterns of children’s social contact and social approach in the classroom. Likewise, additional measures, such as self-reported friendship or social skill ratings, that might have accounted for homophily were not available. Additionally, in two pairs of classrooms, TD children typically attended both morning and afternoon sessions, but those with DD only attended one of the sessions, potentially contributing to increased homophily among TD children in these classrooms. However, supplementary analyses comparing classrooms in which this double attendance pattern was present or not present yielded similar homophily effects in both social approach velocities and social contact (Supplementary Tables 3 and 4). Ultimately, the impact of real-world features of naturalistic interaction (in and out of the classroom setting) on children’s patterns of association remains an important research question.

Two additional areas of investigation may be of interest. All variables used in the current approach involved child dyads. Future research might consider the role of larger groups of children. That is, researchers could ask whether children’s behavior could be most adequately modeled by incorporating information about their tendency to be in social contact with and to approach *multiple peers* simultaneously^30^. Likewise, children’s potential preferences for specific peers, would be an exciting are of future investigation^8^. Additionally, current methods could be broadened to include children’s social approach and social contact with teachers to assess how they facilitate communication between groups of children.

In classroom settings, homophily is evident across a range of ages in a variety of dimensions, including child sex, disability, and academic performance^7,8,14^. Despite its centrality to understanding differences in social movement, little is known about patterns of homophily in the behavior of children with their peers in naturalistic contexts such as the preschool classroom (but see^19,31,32^). The tendency to seek out similar partners was evident in the approach velocities and social contact preferences of TD and DD children. We found that concordant dyads of preschool children interacted with one another at higher velocities and spent greater proportions of time in social contact than discordant dyads, indicating homophilic patterns of movement and colocation at an early age in inclusion classrooms. The results suggest the potential of a new generation of objective measures to quantify strengths and deficits in social interaction in naturalistic contexts.

## Methods

### Participants

Participants were 77 children (*M* = 48.26 months, *SD* = 7.47; 33 female). Of the 77, there were 47 children with DD (13 female) and 30 TD children (20 female). In the DD group, there were 24 children with ASD and 23 children with other disabilities (O/DD) (see Supplementary Table 1). Children were Hispanic (66) and non-Hispanic (11), as well as White (74), Black (2), and multiracial (1). The 77 preschoolers were observed in 8 inclusion preschool classrooms, each of which contained a mean of 11.5 children (*SD* = 3.89). Each child was observed on a mean of 3.52 occasions (*SD* = 1.32); the mean duration of these observations was 138.2 min (*SD* = 63.6). There was a total of 26 classroom observations. Summing over children, 669.95 h of tracked movement/orientation data were collected. Because observations spanned almost the entire school day, except for outdoor play, observed activities included both unstructured and structured play activities, including small group work, arts and crafts, and circle time.

Recruitment and procedures were approved by the University of Miami Social & Behavioral Sciences Institutional Review Board (#20160509), and parents or legal guardians of all participating children provided written informed consent. Parental written consent to the current research was high (95.1% of families consented to data collection). All research was performed in accordance with the guidelines and regulations of the Helsinki committee.

Classrooms employed more and less formal inclusion models, including Learning Experiences and Alternative Program (LEAP) and a reverse mainstream model. The LEAP classrooms consisted of a morning and afternoon half-day session, respectively. In two pairs of classrooms, all but one TD children were present in both the morning and afternoon sessions (see Supplementary Table 1a). Eligibility group was determined by classroom (e.g., dedicated inclusion classrooms) and children’s Individualized Education Programs (IEP) as supplemented, in two cases, by parent report (i.e., report of an ASD diagnosis). Children’s primary category of eligibility for special education services on their IEP determined eligibility group. TD children did not have IEPs.

### Data collection

**Figure 6:**
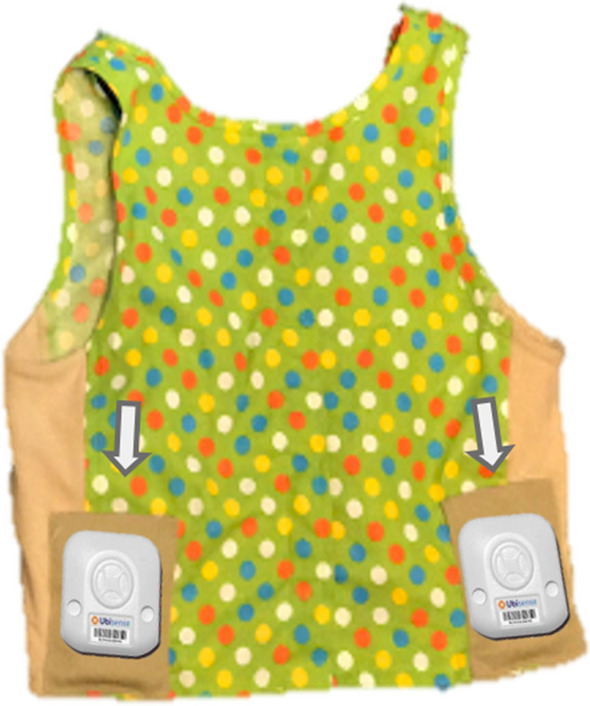
Vest and RFID trackers. Vest with pockets for Ubisense tags (1″ × 1″ × 0.36″).

**Figure 7:**
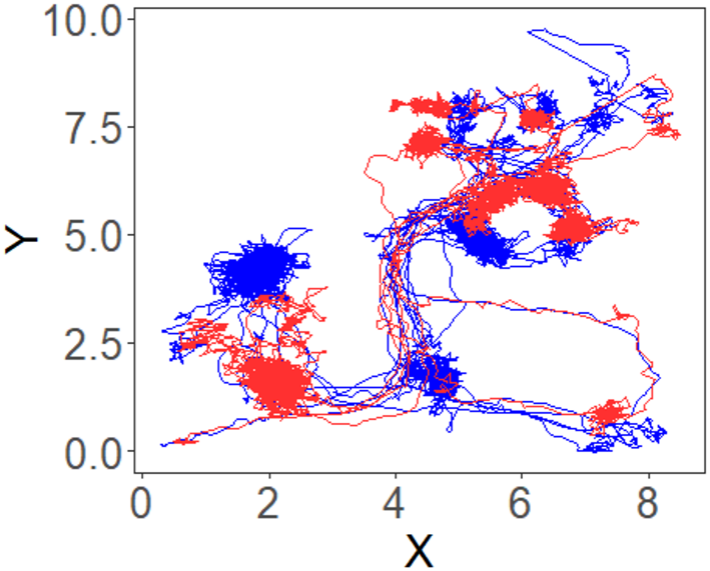
Location trajectory illustration. Trajectory of two children over the course of one half-day session in a preschool classroom as measured by RFID. X and Y refer to the width and length of the classroom in meters.

UWB RFID tracking allowed for automated real-time assessment of children’s location and orientation. These measurements were collected using the Ubisense Tag Module Research Dimension4 with Research Upgrade (see Fig. 6). The Ubisense system tracks children’s positions 2–4 Hz (up to 4 times a second) to an accuracy of 15–30 cm in three-dimensional classroom spaces^24^. Classroom dimensions were 8.97 × 8.86 m., 8.76 × 8.93 m., 9.58 × 8.70 m, and 8.39 × 11.12 m. The classrooms were organized into activity areas, which varied by classroom. Four radio cell sensors (linked by a network cable) in the corners of the classrooms provided data to a dedicated laptop running Ubisense Location Recorder software. The origin (0,0) for the sensor system in each of the classrooms was located at the sensor in the corner closest to the central door. Using RFID, sensors tracked active tags worn by children. Tags were located in space by means of triangulation (angle of arrival, AoA) and time differences in arrival (TDoA). Each child wore two tags (left and right) to provide orientation—the direction being faced—in pockets sewn into a specially-designed vest. The midpoint of the tags’ XY coordinates indexed an individual’s location. Location can be used to visualize individual movement throughout the school day (see Fig. 7). Vests containing RFID tags were placed on children early in the morning by lab personnel to minimize any disruption of the classroom daily schedule. Vests were generally well-tolerated by children (> 97% compliance). Over a total of 271 vest-fitting instances, children refused to wear a vest on 3 occasions. No data were collected on children whose parents did not consent to participation. These children did not wear ultrawide RFID tags.

To account for the possibility of signal loss caused by occlusion (a tag being blocked) or other factors, we utilized linear interpolation to impute missing position data between two known timepoints if data were missing for less than 60s.

### Measures

We present equations to characterize individual and dyadic measures of social interaction in the classroom, using position and orientation as collected by the tags to compute social approach velocities and time in social contact^33^. Tag location data were interpolated for each consecutive tenth of a second. Velocities were computed as differences in location between consecutive tenths of a second and smoothed using a moving average of order 10. Social approach is the mean proportion of the distance between a pair of children traversed by the Approacher per tenth of a second. Time in social contact is the time the pair were in social contact divided by the time both children were present in the classroom. Periods of social contact for each observation were determined using computational modeling tools from statistical physics based on the location and mutual orientation of child–child pairs.

#### Linear velocity

Each child’s position was calculated as the midpoint of their left and right tags. linear velocity (displacement over time) was computed as the difference in position of each child as follows:

1 $$Linear \; velocity\;  [t] =\sqrt{{(x[t]-x[t-1])}^{2}+{(y[t]-y[t-1])}^{2}} $$

where $$x[t]=\frac{1}{2}({x}_{R}[t]+{x}_{L}[t])$$ and $$y[t]=\frac{1}{2}({y}_{R}[t]+{y}_{L}[t])$$.

#### Social approach velocity

To determine social approach velocity, we first calculated the distance each child moved toward or away from the initial position of their partner. Positive and negative values indicate moving toward and away from a partner, respectively.

We begin by defining the distance from child A to partner B at $t$:

2 $$Distance _{A\to B} \; [t-1]=\sqrt{{({x}_{A}[t-1]-{x}_{B}[t-1])}^{2}+{({y}_{A}[t-1]-{y}_{B}[t-1])}^{2}}$$

We define subsequent distance as distance between A’s location at $t$ and B’s location at $t-1:$

3 $$Subsequent \;Distance _{A\to B} \; [t]= \sqrt{{({x}_{A}[t]-{x}_{B}[t-1])}^{2}+{({y}_{A}[t]-{y}_{B}[t-1])}^{2}}$$

Thus, the difference between these distances reflects A’s movement toward B’s previous location:

4 $$ {\text{Approach}}_{{{\text{A}} \to {\text{B}}}} [t] \, = {\text{ Distance}}_{{{\text{A}} \to {\text{B}}}} [t - 1] - {\text{Subsequent Distance}}_{{{\text{A}} \to {\text{B}}}} [t] $$

Social approach involves both an agent (the Approacher) and a partner (the child being Approached). Children with DD and TD children may both differentially approach and be differentially approached by other children. Here, we operationalize homophily in the classroom context as greater velocity of social approach in similar dyads of children. We further ask whether potential differences in the social approach characteristics of children with DD in the preschool classroom are matched by more general difficulties with physical movement^34^. The logic here is to capture A’s movement toward B (while temporarily disregarding any movement of B with respect to A). The approach parameter *Approach*_A→B_ was weighted by A’s orientation to B and the orientation of A’s movement toward B (more direct orientation was weighted more heavily), and A’s distance from B (movement from a closer position was weighted more heavily). Specifically, we utilized θ_o_[$t-1$]—the orientation of the approaching child to the child being approached, θ_*m*_[t]—the angle of movement of the approach relative to the child being approached, and *Distance*_A→B_, initial distance between the pair, to weight *Approach*_A→B_ as follows:

5 $${Social \; Approach}_{Weighted}[t]=\frac{{Approach}_{A\to B}[t]\cdot co{s}^{2}({\theta }_{o}[t-1]) \cdot co{s}^{2}({\theta }_{m}[t])}{{ Distance}_{A\to B}[t-1]}$$

In Eq. (5), orientation angle is used to weight approaches. Higher weights are assigned when A is directly facing B (oriented at 0°) or when A is facing directly away from B (oriented at 180°). These angles characterize A’s instantaneous orientation with respect to B. We are also concerned with the angle of A’s movement with respect to B, which follows an identical logic. That is, direct movement toward or away from B is weighted more heavily than movement perpendicular to B. Finally, approaches from closer locations are weighted more heavily, and approaches from more distant locations are weighted less heavily. This measure of approach velocity is a weighted proportion of the initial distance between the pair. Only positive values of the weighted social approach variable were used in analyses.

#### Time in social contact

**Figure 8:**
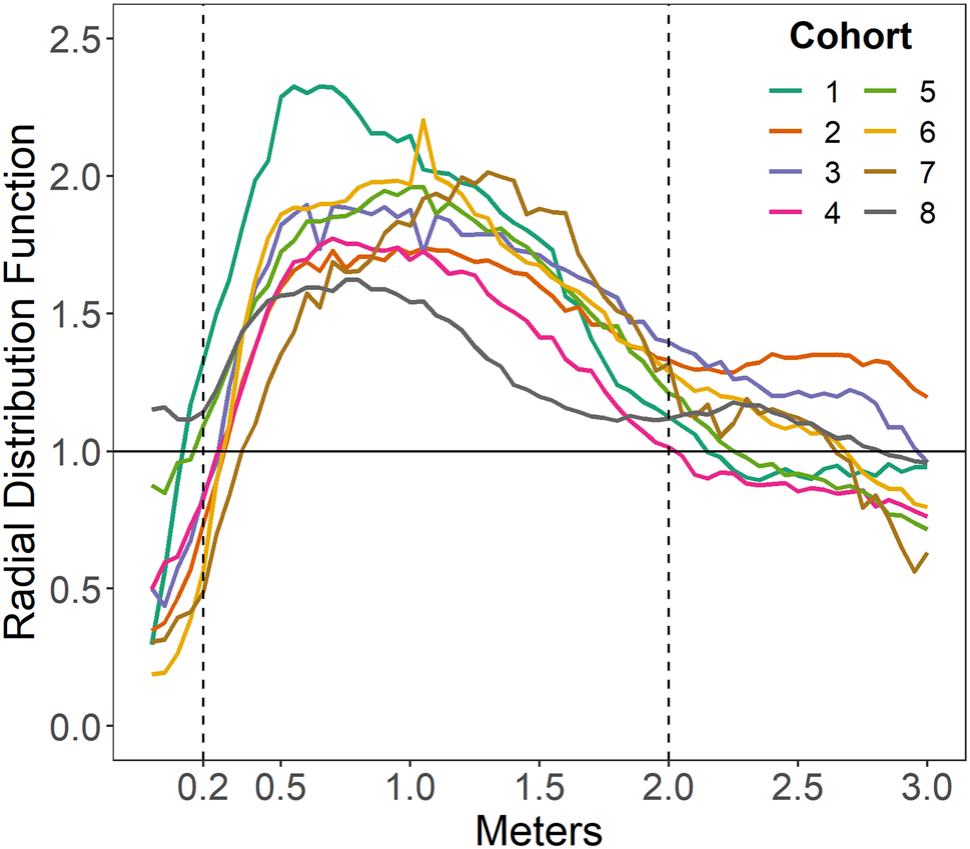
Radial distribution function. The radial distribution function, g(r), is the ratio of the observed distance between children divided by the distance expected by chance. Dyads are determined to be in social contact when children are co-located between 0.2 and 2.0 m and within 45° of one another in relative orientation.

To detect social interaction, the radial distribution function, g(r), indicated when child-child pairs co-locate at closer distances (radii) than expected by chance. Chance refers to the overall location of children during the entirety of an observation irrespective of the positions of others^8,35^. This is a null model of the cumulative probability of a child being in a specific location in the classroom over an entire observation. Using the distances expected by chance, the radial distribution function establishes a distance criterion for two children being in social contact. In addition to the distance criterion there was an orientation criterion for two children to be in social contact. To fulfill the distance criterion, the pairs had to be separated by between 0.2 and 2 m (see Fig. 8). To fulfill the orientation criterion, each child needed to be oriented to the other at less than or equal to |45°|. Social contact was expressed as a unitless proportion of the time when pairs of children were both in the classroom.

### Mixed-effects models

Mixed-effects (multilevel) modeling was conducted in R^36^. Mixed effects regression models were fitted and compared using the lmer function in the “lme4” package. Effect sizes were estimated using the lme.dscore function in the “EMATools” package. Observations (Level-1) were nested in children (Level-2) and children were nested in classroom (Level-3). Models employed restricted maximum likelihood (REML) estimation method for parameterization and model comparison^37^. The significance test of fixed effects was performed using the Wald test^38^. All models contained a random child intercept and a random classroom intercept to account for variance associated with those groupings. The significance of these random effects was estimated by comparing models with and without the corresponding random effect, where differences in model deviance [− 2*(Log Likelihood)] were distributed as chi-square. Between-subjects effects were parameterized as a TD intercept with DD contrasts. Estimated coefficients and effect sizes (Cohen’s *d*) and *t* statistics from final models are presented. In modeling both social approach and social contact, we employed DD versus TD contrasts in addition to contrasting concordant (DD-DD, TD-TD) versus discordant (TD-DD) dyads (see Table 1).

## Supplementary Information

Supplementary Tables.

## Data Availability

The data and R code that support the findings of this study are available on the Open Science Framework: https://osf.io/kftvw/.
