# Supplementary material for: Objective quantification of homophily in children with and without disabilities in naturalistic contexts
Source: Sci Rep. 2023 Jan 17;13:903. doi: 10.1038/s41598-023-27819-6 (PMC9845319; doi:10.1038/s41598-023-27819-6)
Supplement: Supplementary file 1 — Supplementary Tables. [file 41598_2023_27819_MOESM1_ESM.docx]

Objective Quantification of Homophily in Children with and without Disabilities in Naturalistic Contexts

# Chitra Banarjee^1^*, Yudong Tao^2^, Regina M. Fasano^1^, Chaoming Song^3^, Laura Vitale^1^, Jue Wang^4^, Mei-Ling Shyu^2^, Lynn K. Perry^1^, and Daniel S. Messinger^1^*

^1^ Department of Psychology, University of Miami, Coral Gables, FL, USA

^2^ Department of Electrical & Computer Engineering, University of Miami, Coral Gables, FL, USA

^3^ Department of Physics, University of Miami, Coral Gables, FL, USA

# ^4^ Department of Psychology, University of Science and Technology of China, China

# Supplementary Materials

| Supplementary Table 1a. Sample Characterization | | | | | | |
| --- | --- | --- | --- | --- | --- | --- |
| **Classroom** | **Total Children (Girls)** | | | |  | **Teachers** |
|  | **TD** | **ASD** | **O/DD** | **DD** | **Total** |  |
| 1 | 8 (5) | 4 (0) | 0 | 4 (0) | 12 (5) | 3 |
| 2 | 8 (5) | 3 (0) | 0 | 3 (0) | 11 (5) | 4 |
| 3 | 7 (6) | 4 (1) | 0 | 4 (1) | 11 (7) | 2 |
| 4 | 8 (7) | 3 (1) | 0 | 3 (1) | 11 (8) | 2 |
| 5 | 1 (0) | 3 (1) | 7 (0) | 10 (1) | 11 (1) | 2 |
| 6 | 1 (0) | 1 (0) | 4 (2) | 5 (2) | 6 (2) | 2 |
| 7 | 10 (7) | 4 (1) | 6 (3) | 10 (4) | 20 (11) | 2 |
| 8 | 2 (1) | 2 (1) | 6 (3) | 8 (4) | 10 (5) | 2 |

*Note*. The DD column is the sum of the ASD and O/DD columns. There were four classrooms in which children with ASD were observed either during the morning or afternoon, while TD children typically remained in the classroom for the entire day. Thus, the 8 TD children in Classroom 1 and Classroom 2 are the same children. Likewise, 7 of the 8 TD children in Classroom 4 are also in Classroom 3. There were 12 female teachers (Hispanic (9), non-Hispanic (3), White (10), and Black (2)), with 2-3 in each classroom.

Supplementary Table 1b. Age and Preschool Language Scales-5 (PLS-5) Standardized Scores

|  | **Age (SD) in months** | **AC Standard** | **EC Standard** | **Standard Language Score** |
| --- | --- | --- | --- | --- |
| TD | 82.51 (8.83) | 113.41 (16.32) | 111.28 (22.42) | 113.24 (2.36) |
| ASD | 81.26 (11.24) | 82.45 (17.02) | 74.4 (16.42) | 77.15 (16.58) |
| O/DD | 82.27 (8.45) | 92.18 (16.54) | 82.82 (18.65) | 86.68 (17.32) |
| DD | 81.75 (9.88) | 87.55 (17.28) | 78.81 (17.93) | 82.14 (17.45) |

*Note*. PLS-5 assessments were administered at the beginning of the school year for the 8 classrooms. The table presented here are the mean ages (in months) for each group and the mean (standard deviation) auditory comprehension (AC), expressive communication (EC), and standard language scores for each group. The DD row aggregates the ages and scores for the ASD and O/DD groups. Out of the 77 total children, 6 children did not receive language characterization.

| Supplementary Table 2. Social Approach – Full Model | | | | | | |
| --- | --- | --- | --- | --- | --- | --- |
|  | Social Approach | | | | | |
| *Predictors* | B | SE | CI | *t* | *p* | *d* |
| (Intercept) | .00317 | .00023 | .00272 – .00363 | 13.68 | **<.001** |  |
| Approacher [DD] | -.00006 | .00008 | -.00021 – .00010 | -.71 | .479 | -.16 |
| Approached [DD] | -.00009 | .00004 | -.00018 – -.00001 | -2.12 | **.034** | -.08 |
| Homophily [Concordant] | .00020 | .00004 | .00012 – .00028 | 4.77 | **<.001** | .17 |
| Random Effects | | | | | | |
| σ^2^ | .00000 | | | | | |
| Child | .00000 | | | | | |
| Classroom | .00000 | | | | | |
| ICC | .29749 | | | | | |
| Observations | 3108 | | | | | |

*Note.* Social approach is the mean proportion of the distance between a pair of children traversed by the Approacher. The full model contains Approacher (the child who is approaching), Approached (the child being approached), and Homophily (if the members of the dyad are concordant or discordant) terms. The final model presented in Table 1 removes the nonsignificant Approacher term. DD includes both ASD and O/DD.

| Supplementary Table 3. Social Approach – Class Type Model | | | | | | |
| --- | --- | --- | --- | --- | --- | --- |
|  | Social Approach | | | | | |
| *Predictors* | B | SE | CI | *t* | *p* | *d* |
| (Intercept) | .00348 | .00024 | .00300 – .00395 | 14.29 | **<.001** |  |
| Approached [DD] | -.00005 | .00004 | -.00013 – .00004 | -1.03 | .303 | -.04 |
| Homophily [Concordant] | .00034 | .00006 | .00022 – .00047 | 5.46 | **<.001** | .22 |
| ClassType  [non-LEAP] | -.00073 | .00034 | -.00141 – -.00006 | -2.13 | **.033** | -1.71 |
| Homophily *  ClassType | -.00025 | .00009 | -.00042 – -.00008 | -2.91 | **.004** | -.11 |
| Random Effects | | | | | | |
| σ^2^ | .00000 | | | | | |
| Child | .00000 | | | | | |
| Classroom | .00000 | | | | | |
| ICC | .20937 | | | | | |
| Observations | 3108 | | | | | |

*Note.* Social approach is the mean proportion of the distance between a pair of children traversed by the Approacher. This model contains a variable, ClassType, that distinguishes classrooms 1-4 (LEAP classrooms), where all but one TD children were present in both the morning and afternoon sessions. σ^2^ - residual variance at level 1 (observation). ICC- intraclass correlation. Approached [DD] compares velocities at which children with DD were approached to velocities at which children with TD were approached. ClassType compares non-LEAP classrooms to LEAP classrooms. DD includes both ASD and O/DD.

| Supplementary Table 4. Social Contact – LEAP Model | | | | | | |
| --- | --- | --- | --- | --- | --- | --- |
|  | Time in Social Contact | | | | | |
| *Predictors* | *B* | SE | CI | *t* | *p* | *d* |
| (Intercept) | .04 | .00 | .03 – .05 | 8.27 | **<.001** |  |
| [DD] | .00 | .00 | -.00 – .01 | .14 | .891 | .02 |
| Homophily [concordant] | .02 | .00 | .01 – .02 | 7.69 | **<.001** | .28 |
| ClassType  [nonLEAP] | .01 | .01 | -.01 – .02 | 1.09 | .274 | .79 |
| [DD] * Homophily  [concordant] | -.01 | .00 | -.02 – -.00 | -2.57 | **.010** | -.09 |
| [DD] * ClassType | -.00 | .00 | -.01 – .01 | -.08 | .933 | -.01 |
| Homophily * ClassType | -.01 | .00 | -.02 – -.01 | -4.38 | **<.001** | -.16 |
| [DD] * Homophily * ClassType | .01 | .01 | -.00 – .02 | 1.10 | .271 | .04 |
| Random Effects | | | | | | |
| σ^2^ | .00 | | | | | |
| Child | .00 | | | | | |
| Classroom | .00 | | | | | |
| ICC | .09 | | | | | |
| Observations | 1644 | | | | | |

*Note*. Time in social contact is the time the pair were in social contact divided by the time both children were present in the classroom. This model contains a variable, ClassType, that distinguishes classrooms 1-4, where all but one TD children were present in both the morning and afternoon sessions. σ^2^ - residual variance at level 1 (observation). ICC- intraclass correlation. The [DD] term compares the proportion of time in social contact for children with DD with time in social contact for children with TD. The Homophily term indicates that children spent greater proportion of time in social contact when interacting with a concordant partner, compared to a discordant partner. The interaction term indicates that children with DD spent less time in social contact with other DD children, compared to TD-TD dyads. ClassType compares non-LEAP classrooms to LEAP classrooms. DD includes both ASD and O/DD.

| Supplementary Table 5. Linear Velocity | | | | | | |
| --- | --- | --- | --- | --- | --- | --- |
|  | Linear Velocity (cm/sec) | | | | | |
| Predictors | *B* | SE | CI | *t* | *p* | *d* |
| (Intercept) | 31.06 | 1.63 | 27.87 – 34.25 | 19.11 | <.001 |  |
| [DD] | -.20 | .63 | -1.44 – 1.04 | -.31 | .754 | .07 |
| Random Effects | | | | | | |
| σ^2^ | 17.46 | | | | | |
| Child | 1.63 | | | | | |
| Classroom | 19.56 | | | | | |
| ICC | .55 | | | | | |
| Observations | 314 | | | | | |

*Note.* The [DD] term compares linear velocity, overall speed of movement, between DD and TD children, finding no significant differences in the individual measure of movement. DD includes both ASD and O/DD.
